# Factorial Validity and Variance of the Maslach Burnout Inventory between Demographic and Workload Groups among Dentists of Lithuania

**DOI:** 10.3390/ijerph17249154

**Published:** 2020-12-08

**Authors:** Eglė Slabšinskienė, Andrej Gorelik, Ingrida Vasiliauskienė, Aistė Kavaliauskienė, Apolinaras Zaborskis

**Affiliations:** 1 Department of Oral Health and Paediatric Dentistry, Faculty of Odontology, Medical Academy, Lithuanian University of Health Sciences, A.Mickevičiaus 9, LT-44307 Kaunas, Lithuania; andrej.gorelik@gmail.com (A.G.); ingrida.vasiliauskiene@lsmuni.lt (I.V.); 2 Department of Orthodontics, Faculty of Odontology, Medical Academy, Lithuanian University of Health Sciences, A.Mickevičiaus 9, LT-44307 Kaunas, Lithuania; aiste.kavaliauskiene@lsmuni.lt; 3 Department of Preventive Medicine & Health Research Institute, Faculty of Public Health, Medical Academy, Lithuanian University of Health Sciences, A.Mickevičiaus 9, LT-44307 Kaunas, Lithuania; apolinaras.zaborskis@lsmuni.lt

**Keywords:** burnout, dentist, workload, Maslach burnout inventory, factorial validity, Lithuania

## Abstract

There is a lack of evidence about burnout syndrome among Lithuanian dentists; as a reliable instrument to examine the syndrome in this professional group has not yet been tested. The study aimed to investigate the performance of the items and the subscales of the Maslach burnout inventory (MBI) by validating its factorial structure and analyzing its variance between demographic and workload groups of dental professionals in Lithuania. The survey was conducted among practicing dentists online or during the scientific conferences for dentists using an anonymous questionnaire. To evaluate the level of burnout the MBI was chosen. Reports of a total of 380 respondents were examined. Three-factor structure of the MBI with cross-loading of two items suggested a good fit to data (χ^2^/df = 1.67; CFI = 0.93; TLI = 0.93; IFI = 0.93; and RMSEA = 0.059) and was invariant across demographic and workload groups of dentists. Multigroup factorial analysis revealed that females as compared to males had higher average emotional exhaustion; and the respondents up to 30 years as compared to respondents over 30 years of age had higher averages of the emotional exhaustion and depersonalization components. Highly specialized dentists (oral surgeons, prosthodontists, orthodontists, endodontists and poedodontists) were particularly less prone to burnout syndrome than dentists of general practice. It was concluded that the MBI offers factorial validity and demonstrates its invariant structure and variance of burnout dimensions across demographic and workload groups. These findings are informative for burnout prevention and intervention programs among dentists in Lithuania. Such information may contribute to lessen professional burnout among dentists in Lithuania.

## 1. Introduction

Job burnout in healthcare professionals has become a challenge for researchers around the globe during the past few decades [1,2,3]. Various theoretical models and research studies from many countries have contributed to a better understanding of the causes and consequences of this professionally specific disorder of well-being and ability to work [4,5,6,7]. Numerous systematic reviews and meta-analyses on burnout of physicians and nurses have been published currently [8,9,10,11].

Burnout is a highly complex phenomenon whose origins are multifactorial, so measuring it is a challenge for researchers. Without doubt, the most common instrument to determine the level of burnout in healthcare and other professionals is Maslach burnout inventory (MBI), considered to be the “gold standard” of this type of measurement first published in 1981 [12] and later modified to fit different groups of respondents [13]. This instrument is comprised of 22 items, each scored from 0 to 6 based on self-reported frequency of the feeling addressed by each item. Although there are different opinions on the dimensional structure of the questionnaire, the MBI authors described burnout as a three-dimensional construct of emotional exhaustion (EE), depersonalization (DP) and reduced personal accomplishment (PA), which consist of 9, 5 and 8 items respectively. Using data from U.S. samples, Maslach’s team demonstrated that these dimensions have good psychometric properties [12]. In order to evaluate the level of burnout prevalence, researchers are encouraged to use the cut-off points defined by the mean value and standard deviation of the MBI summed score within the sample itself [13].

The MBI instrument has already been translated into Lithuanian, validated and used in the professional burnout studies in Lithuania among hospital physicians [14], neonatal nurses [15], anesthetists and intensive care physicians [16]. However, a similar study has not yet been conducted among the dental profession.

The dental profession in Lithuania has gone through deep and extreme organizational changes in the last decades [17]. After the restoration of Lithuania’s independence and with the introduction of the free market in the country dentistry was one of the first health care sectors that very rapidly became predominantly driven by the private sector. In essence, the dental profession in Lithuania has lost a special status it held among other medical professions. A general practicing dentist started to get multiple stresses while carrying out clinical, administrative and managerial tasks. Being a free entrepreneur, commercial aspects play a significant role in dentistry, while at the same time there are strong restrictions and control by government and sanitary institutions. Moreover, dentistry is a very demanding profession with ever-changing technologies and methods of practices, with high stress and high expectations from self and patients. The accumulative results of this stress leads to burnout [18,19]. Studies have shown that dentists were significantly more likely to experience burnout symptoms than any other medical profession group [20].

Many studies have been done on burnout of dentists in other countries [18,21,22,23], but none relate the burnout of Lithuanian dentists. A review of the literature makes it possible to conclude that the MBI has been employed with the greatest frequency to measure the burnout syndrome in dentistry. To perform similar research among Lithuanian dentists, we sought to ascertain how valid and reliable the MBI is in the country’s dental population with respect to comparing results between countries and cultures.

Modern research in factorial validity includes exploratory factor analysis (EFA) and confirmatory factor analysis (CFA) seeking for the best fit of the models to available data. In relation to studies that have examined the MBI 22-item versions, the number of factorial analysis studies [24,25,26,27] has shown a three-factor structure, representing EE, DP and PA components firstly introduced by authors [12]. Nevertheless, other studies have showed some weakness of the MBI related to factorial validity. Some of these studies reported good fit for a two-factor [28,29], or a four-factor [30] and a five-factor structure [31]. Furthermore, the studies have consistently forced to load each item on the target factor without allowing the cross-loading of items on non-target factors, and that causes inflation of the estimated factor correlation [32]. So far, in some studies among dentists the original MBI was used, but no testing of the factorial structure of the instrument used was reported [27,33]. It is likely that the heterogeneity of the findings is due to the fact that the sample sizes in most studies were less than optimal for conducting factor analysis, and in some of the larger sample studies certain items were removed from the MBI [26,34].

Important psychometric characteristics of an instrument such as MBI that accompany analysis of the factorial validity are internal consistency (reliability), construct validity and convergent validity [35,36]. Internal consistency estimated by Cronbach’s alpha in the original MBI study was 0.90 for EE subscale, 0.79 for DP subscale and 0.71 for PA subscale [12]. Further studies showed similar figures, but poor internal consistency coefficients for the DP subscale were found in most of these studies [24,37]. In regard to factorial models, testing of construct validity is employed to determine the MBI factorial model’s invariance across subgroups of respondents, and testing of convergent validity investigates the variation of the MBI dimensions among subgroups of respondents. Only a few empirical studies have been published on testing these important psychometric characteristics. Bria et al. [36] tested models’ invariance and the variance of the MBI dimensions across different occupational and demographic subgroups of healthcare professionals by means of multigroup analysis; results confirmed the expected values of these psychometric characteristics for the three-factor MBI model. Following the example of this study, it is worth investigating the invariance of the MBI model and the variation of its dimensions among dentists, which, to our knowledge, has not yet been done. In such a study, the gender, age and workload factors should be considered first.

The purpose of the present study was to investigate the performance of the items and the subscales of the Maslach burnout inventory (MBI) by validating its factorial structure and analyzing its variance between demographic and workload groups of dental professionals in Lithuania.

## 2. Materials and Methods

### 2.1. Sample Size Calculation

Statisticians suggest the sample size for factor analysis equal to 300 is good or suggest the optimal respondent-to-item ratio 10:1 [35,38] (in the present study 220 respondents for a 22-item questionnaire). As larger samples are always better than smaller samples, it was decided to collect data from at least 300 respondents. Assuming that only about 60 percent of the dentists in the initial sample would participate in the study, the sample size was increased to 550. The license to reproduce the required number of MBI copies (400 hard-copies and 150 online-copies) was purchased from the copyright holder (Mind Garden, Inc., USA).

### 2.2. Study Design, Participants and Data Collection

**Figure 1:**
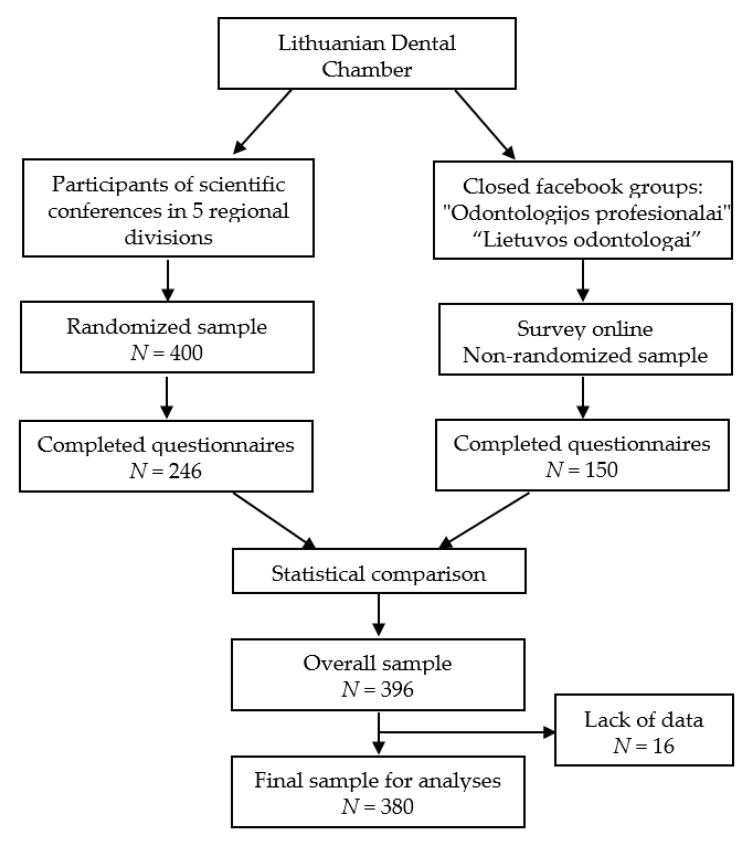
Flow chart of the data collection process.

This observational study had a cross-sectional design. Data collection was performed, by in two ways (Figure 1). In the first way, the survey was conducted among dentists who attended scientific conferences organized by the Lithuanian Dental Chamber in five regional divisions in October–December, 2019. Attendance of these conferences is regulated by the licensing conditions for dentists and supported by the Lithuanian Dental Chamber, therefore, participation in these events is active among dentists in all regions of Lithuania. With the consent of the conference organizers, 400 dentists were randomly selected (80 attendees at each conference) and at the registration desk were invited to fill in the hard-copy questionnaire. Of them, 246 (61.5%) dentists agreed to participate in the study and completed the questionnaire. As the number of subjects was still insufficient, data collection was continued in a second way by conducting an online survey in January–February, 2020. The questionnaire was shared in closed Facebook groups “Odontologijos profesionalai” (“Professionals of Odontology”) and “Lietuvos odontologai” (“Dentists of Lithuania”) with limited access destined only to its members. Those who completed the questionnaire during the conferences were informed not to complete it. In this way, another 150 completed questionnaires (all purchased online copies) were received, however, the response rate for this group of respondents remained unknown. Altogether, 396 dentists participated in the study. So, this sample was comprised of two groups. The first (“conference”) group, which was larger (246 subjects or 62% of the total sample), can be considered as a randomized subsample, while the second (“on-line”) group is a non-randomized subsample, as its participant pool may not include every dentist of the country. Comparison of these groups according to demographic and other factors used in the study did not show statistically significant differences, so it was decided to analyze the whole sample.

### 2.3. Burnout Measure

To evaluate levels of burnout among dentists MBI-HSS (Maslach burnout inventory—Human Services Survey) was chosen as it is considered to be the most trustworthy and the most commonly used tool of this kind [13]. We adapted it for dentists who belong to a specific group of health professionals.

The instrument was translated into Lithuanian and was validated previously in other fields of medicine [14,15,16]. It comprised 22 items (V1 to V22; list of items can be seen in Results section). The responses were ranked on a 7-point Likert scale from 0 (“never”) to 6 (“daily”). In analyses, the items V4, V7, V9, V12, V17, V18, V19 and V21 were reverse-scored, hence higher item scores implied higher level of burnout (higher emotional exhaustion and depersonalization and lower personal accomplishment).

### 2.4. Statistical Analysis

The data were analyzed using the SPSS (version 21.0; SPSS Inc., Chicago, IL, USA, 2012) statistical package supplemented with AMOS [39].

Descriptive statistics were employed to calculate means, medians and standard deviations of the continuous variables, and to calculate percentages of the categorical data. The Kolmogorov–Smirnov one-sample test was used to assess whether the scales’ summed scores were normally distributed. The cut-off level for statistical significance was set at 0.05.

In order to understand the interrelations among the items and to confirm the inherent structure of the MBI, an exploratory factor analysis (EFA) was performed. The appropriateness of the models was evaluated with the Kaiser–Meyer–Olkin (KMO) measure along with the Bartlett’s test (KMO ≥ 0.5 and *p* < 0.001 show the adequacy of the data for use in the EFA). We carried out a principal components factor analysis with a Promax rotation in which the factors are assumed to be correlated. Initially, factors were extracted based on the break point of successive eigenvalues (≥1), then the number of factors was limited based on the interpretability of the results. According to Hair et al. [40], only standardized factor loadings greater than 0.5 were taken in account.

Second, a confirmatory factor analysis (CFA) [39,41,42] was conducted to investigate the model fitness based on the EFA findings. We reported several goodness-of-fit indicators: the relative Chi-square (χ^2^/df), comparative fit index (CFI), Tucker–Lewis index (TLI), Bollen’s incremental fit index (IFI) and root mean square error of approximation (RMSEA). A value of χ^2^ divided by the degrees of freedom (χ^2^/df) between 1.0 and 3.0 may be considered acceptable goodness-of-fit but it is highly sensitive to sample size and number of constraints [41,42]. Further, the CFI, TLI and IFI are Chi-square-based calculations independent of degree of freedom; the recommended thresholds for these values are ≥0.90. The RMSEA tests the fit of the model to the covariance matrix, therefore it was considered as the main criterion. The majority of researchers consider that RMSEA values lower than 0.05 indicate a very good fit and value up to 0.08 signals a reasonable fit [42]. The CFA was realized with a software AMOS (version 21.0; SPSS Inc., Chicago, IL, 2012) [39].

Regarding the generalizability of the findings, factor analysis was performed by randomly splitting the entire sample into two subsamples [43]. Both subsamples included an equal number of subjects (*N* = 190). Statistical comparisons of demographic and workload factors between subsamples was conducted; there were no statistical differences between subsamples. Subsequently, subsample 1 was used for EFA to examine the structure of the MBI in the dentists’ sample, and subsample 2 was used as a validation sample for the identified structure from the EFA and CFA was adopted. The further multiple-group analysis of invariance and multiple-group analysis of factor means were conducted in the entire sample (*N* = 380).

A set of tests was used for examination of psychometric properties of the MBI [35,36]. The Cronbach α and McDonald’s ω were used as a measure of internal consistency of the total scale considering coefficient ω as a more robust estimation for a response scale with correlated errors [44]. Furthermore, other tests of internal reliability (inter-item and item-total correlations) were also investigated.

### 2.5. Ethical Statement

The study conformed to the principles outlined in the World Medical Association’s Declaration of Helsinki. It was approved by the Bioethics Committee Center of the Lithuanian University of Health Sciences on 16 October, 2019 (Protocol number: BEC–OF–13). The confidentiality and anonymity of the participants was guaranteed.

## 3. Results

### 3.1. Sample Characteristics

We excluded subjects with a lack of data on sample characteristics or the MBI (*n* = 16, 4.0% of the original sample of 396). Finally, a total of 380 subjects were left for analyses in this study. Profile of demographic and workload characteristics of these respondents are given in Table 1. Most of the participating dentists were females (84.7%). Respondents ranged in age from 23 to 80 years, with a mean age of 37.3 (SD 12.9) years for the study sample. So, almost half of the dentists (46.3%) have work practice experience of 10 or more years. There were also a variety of subjects according to the workload characteristics of interest (staffing and number of working places). Although most of the dentists in the study were dentists in general practice (73.3%), small groups of dentists from other specialties also participated in the study.

### 3.2. Descriptive Characteristics of the Maslach Burnout Inventory

There was a good overall response rate to the items on the scale. In fact, 14 (3.5%) respondents of the total number (*N* = 396) of dentists who participated in the survey left blank 5 or more items of the scale, they were excluded from the further analysis; for the remaining records, missing data were corrected with an individual average value.

Table 2 presents percentage distribution of dentists’ burnout level for all items of MBI. Majority of dentists were positive about their patients. For example, a high percentage of respondents rated “daily” or “several times a week” the following items: V4 “I can easily understand how my recipients feel about things” (81.2%); V7 “I deal very effectively with the problems of my recipients” (72.4%); V9 “I feel I’m positively influencing other people’s lives though my work” (77.9%); V17 “I can easily create a relax atmosphere with my recipients” (71.9%) and V19 “I have accomplished many worthwhile things in this job” (63.9%). Most dentists expressed their personal fulfillment choosing the answer “never” or “a few times a year” to rate the items: V5 “I feel I treat some patients as if they were impersonal objects” (66.0%); V10 “I’ve become more callous toward people since I took this job” (61.8%) and V15 “I don’t really care what happens to some patients” (82.7%). The top 3 items according to the percentage distribution of the respondents’ rating “a few times a week” or “every day” (considered to be have high impact on the burnout level) were V2 “I feel used up at the end of the workday” (46.3%), V1 “I feel emotionally drained from my work” (38.4%) and V6 “Working with people all say is really a strain for me” (35.0%).

It was found a normal distribution of summed score (one-sample Kolmogorov–Smirnov test *p* = 0.268). Its values ranged from 4 to 104, with a mean of 44.5 (a median of 44) and a standard deviation of 20.2. There was a tendency of lower mean for males than for females (40.6 ± 18.5 vs. 45.2 ± 20.5, *p* = 0.111) and significantly higher for younger dentists than for older dentists (48.6 ± 18.6 vs. 42.0 ± 20.9, *p* = 0.002). These results indicate variability of the MBI values.

### 3.3. Psychometric Characteristics

Both measures of internal consistency reliability of the total MBI resulted in almost identical values (Cronbach’s α = 0.895 and McDonalds ω = 0.898) indicating a good internal consistency. The further analysis of the instrument reliability showed that the item V13 “I feel frustrated by my job” is the “strongest” item in the instrument (item-total correlation 0.712 and Cronbach’s α if item deleted 0.886), and the item V4 “I can easily understand how my patients feel about things” is the “weakest” item in the instrument (item-total correlation 0.175 and Cronbach’s α if item deleted 0.898).

### 3.4. Exploratory Factor Analysis

On the first subsample, KMO and Bartlett’s tests indicated that the data were suitable for factor analysis (KMO = 0.867, *p* < 0.001). Primarily, we conducted factor analysis with principal components and Promax rotation to determine the overall factor structure of the MBI. Analysis of the eigenvalues and scree plot indicated that four factors could be extracted with values above 1.0 (6.84, 3.05, 1.33 and 1.20). The four-factor model accounted for 56.44% of the MBI-item variance. Table 3 presents the factor loadings for this solution. All items had salient (≥0.50) loadings on the four factors (range from 0.511 to 0.786). The first factor (F1) combined 10 items (1, 2, 3, 6, 8, 12, 13, 14, 16 and 20); the second factor (F2) combined 7 items (4, 7, 9, 17, 18, 19 and 21); the third factor (F3) combined 3 items (5, 10 and 11) and the fourth factor (F4) combined 2 items (15 and 22). Factorial analysis on the second subsample confirmed the same factor structure (results not presented).

With regard to interpretability, the four-item factor solution seemed to be complicated. Moreover, the fourth factor combined only two items, while the internal consistency of such a subscale was extremely not satisfactory (Cronbach’s α = 0.373). Therefore, the three-factor model was investigated. This model accounted for 50.38% of the total item variance. Table 3 also presents the factor loadings for this solution. Compared to the four-factor model, it can be seen that there were significant changes only in F3 and F4 factors: item 11 went to F1 and both items (15 and 22) of the previous F4 factor were included into F3. Both four-factor and three-factor models have the same property that some items (10, 11, 12, 13 and 14) have sufficiently large (>0.5) and almost equal loadings for two factors. Therefore, this property must be taken into account when constructing the final MBI model. As the structure of the three-factor model was very similar to the composition of subscales provided by the MBI authors [12], extracted factors could be interpreted as follows: F1—emotional exhaustion (EE), F2—personal accomplishment (PA), F3—depersonalization (DP).

### 3.5. Confirmatory Factor Analysis

**Figure 2:**
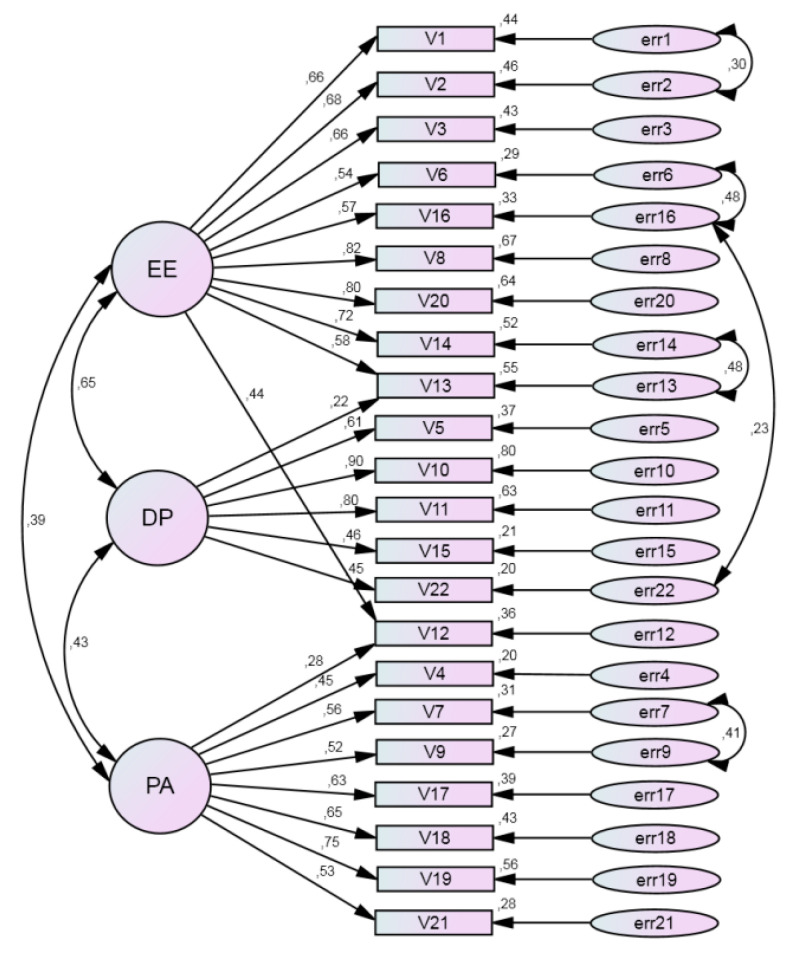
Path diagram with standardized estimates of the factorial structure for the final Maslach burnout inventory model (confirmatory factor analysis, *N* = 190). EE (emotional exhaustion), DP (depersonalization) and PA (personal accomplishment) are common factors; V1, ..., V22 are the Maslach burnout inventory items; err1, ..., err22 are residual errors.

The dimensionality of the MBI was confirmed by the CFA using a randomly selected second subsample of the entire sample. The analysis began with an examination of the three-factor model found in EFA. In this model, there were four cross-loadings: items 10, 11 and 13 were linked with both EE (F1) and DP (F3), and item 12 was linked with both EE (F1) and PA (F2) as corresponding loadings were greater than 0.5 (see Table 3). However, standardized estimates of this model signaled that items 10 and 11 had low and statistically insignificant loading on factor EE. The model fitted the data poorly. Results of the CFA of the model that directly replicated the EFA model are presented in Table 4. Based on these findings the model was revised to achieve a better goodness of fit. First, the link of items 10 and 11 with EE was deleted. Second, examination of modification indices indicated improvement in the fit of the model if several pairs of residual errors were allowed to correlate. In consequence, the revised model included all 22 items of the MBI, in which 8 items were loaded on the factor EE, 7 items were loaded on the factor PA and 5 items were loaded on the factor DP only one at a time, while item 12 (“I feel very energetic”) and item 13 (“I feel frustrated by my job”) remained loaded on two factors. Both items had greater loadings on factor EE (0.44 and 0.58) than on other factors (0.28 on factor PA and 0.22 on factor DP correspondingly for items 12 and 13). Figure 2 demonstrates a path diagram with standardized estimates of the final CFA model (*N* = 190). All factor loadings (the path coefficients leading from the common factors to the observed variables) were found to be significant.

The fit indices for the final model suggested a good fit of data to the model: χ^2^/df = 1.67, CFI = 0.92, TLI = 0.93, IFI = 0.93 and RMSEA = 0.059. The factors were moderately correlated; *r* ranged from 0.39 to 0.65 (see Table 4).

### 3.6. Multiple-Group Analysis of Invariance

Multiple-group analysis was performed to test if the final revised MBI model is invariant across groups of respondents. The focus was on these groups: randomly selected two subsamples, gender (males and females), age (up to 30 and 30 or more year-old) and staffing (half-time and full-time) groups. First at all, we tested if the path diagrams are identical across groups (no cross-group constraints for parameters). Then, constraints on constant factor loadings were included. Finally, constant factor variances and covariances were requested to be constant across groups. Results of analyses are presented in Table 5. There appears to be no significant evidence that model parameter values differ across all tested groups. In analyses, CFI and IFI values varied between 0.90 and 0.92 and TLI between 0.89 and 0.91, while RMSEA values varied between 0.041 and 0.049, which all indicate a good fit of data to the model. Furthermore, at each step the increase in likelihood ratio chi-square statistic was never much larger than the increase in degree of freedom, therefore, the data do not depart significantly from any of the models.

### 3.7. Multiple-Group Analysis of Factor Means

In the next step of multiple-group analyses we tested the null hypotheses that factors EE, PA and DP have the same averages across groups of respondents. Thus, we sought to find out how the averages of these common factors depend on demographic (gender and age) and workload variables (tenure, staffing, number of working places and specialty). Results of this analysis are displayed in Table 6. Females as compared to males provided a greater average EE component, so they were more likely to report more often emotional exhaustion in their work (items 1, 2, 3, 6, etc.). However, there was no gender difference in averages of DP and PA components. Respondents in the younger (up to 30 years of age) group had higher averages of the EE and DP components compared to respondents in the older (30 years of age and older) group. A similar figure was found when comparing groups of respondents by tenure, namely, dentists with shorter (up to 10 years) work practice experience were prone to deeper emotional exhaustion and depersonalization than their colleagues with longer work practice experience. As expected, dentists working full-time (40 hours per week or more) reported higher burnout item rates than dentists working half-time, but this was significant only in the EE and DP, but not in the PA dimensions of the MBI. In contrast, it was found no significant difference in burnout estimations between dentist who work in one clinic and who work in a few clinics.

We were also able to compare the level of burnout syndrome between dentists of different specialization, even if the number of respondents in some specialty groups was small. Compared to the dentists in general practice, highly specialized dentists (oral surgeons, prosthodontists, orthodontists and endodontists) were particularly less prone to burnout syndrome. Poedodontists can also be admitted to this group, as their MBI mean estimates in the DP and PA quite significantly differed from the corresponding estimates of the reference group. However, periodontists were as prone to burnout as there were general practitioner dentists.

## 4. Discussion

The findings of the present study suggest that as a whole the Maslach burnout inventory presents an adequate factorial validity and its three dimensions demonstrate sufficient variance of burnout dimensions between demographic and workload groups among dentists in Lithuania.

The majority of studies on the issue of professional burnout have been related to the methodical use of the MBI, however, such studies often offered divergent results on factorial validity. In samples of health professionals, the three-factor structure of the MBI was driven and conditioned as a standard [24,25,26,27]. Our survey also revealed three-factor structure, but our findings were more in line with the alternative studies, which suggested that the initial three-factor structure could have a better fit to empirical data if several items of the inventory would be excluded [26,34,45], or if some items would be allowed to load on different dimensions than those hypothesized in the standard model [46]. Other studies reported findings of a two [28,29], or a four-factor [30] and a five-factor structure [31]. Good fit for a two-factor model with EE and DP merged into one dimension was also reported [29].

Although dentistry is considered a field with high risk of professional burnout [18], very few empirical studies have been published on burnout for the general dental community [23]. These studies differ in both instruments used and presentation of results. In some of these studies the original MBI was used, but in most cases no testing of the factorial structure of the instrument used was reported [27,47]. In other cases the original scales were completely rewritten without psychometric explanation [48].

Our study was the first in Lithuania that addressed the job burnout in the representative sample of dentists in practice. The findings of study were based on the data of survey that was conducted among practicing dentists online and during the scientific conferences for dentists using hard copies of the questionnaire. Both groups did not differ significantly by the main demographic and research characteristics. Almost 85% of the total sample was females, and this proportion match well the gender distribution of the dentist population in Lithuania (percentage female 83%) [49]. We did not limit the age of the senior participants on the basis of the constitutional provision not to discriminate against employee age, so the sample also included respondents of respectable age. The sample was also representative to the Lithuanian dentists’ trade union by specialties (dental specialists comprise about 17% of the total number of dentists in Lithuania [49]). Therefore, these facts on representativeness of the study participants to the Lithuanian dentist population by demographic and professional characteristics testify to the validity of empirical data.

Results from this study indicated a good internal consistency and factorial validity of the MBI. The final three-factor model of this inventory was invariant across tested groups of respondents. Therefore, it was concluded that variance of its three dimensions could be associated with the demographic and workload predictors. However, because some items of the MBI were related to different factors, we could not definitely classify the items into three separate dimensions, consequently we could not calculate their summed scores and evaluate their relationship with other factors. Instead, we conducted a multiple-group analysis with the CFA to examine the variance of factor means across the subgroups of respondents. From this analysis we revealed several findings relating the three burnout dimensions to gender, age and relevant occupational predictors.

In our study, female dentists had significantly higher EE mean than male dentists, but a significant difference between genders was not observed for means of DP and PA dimensions. Maslach and Jackson [12] found differences between males and females for each of the MBI subscales: females scored higher than males on EE, but males scored higher than females on DP and PA. The authors explained this fact as a role difference, as women being in giving roles, eventually makes them susceptible to emotional exhaustion [12]. Moreover, some studies identified that male dentists have a higher risk of burnout, as they tended to work more hours a week than females [21,27]. Therefore, further studies that control gender are required to find out the multifactorial nature of burnout syndrome.

Values of the EE and DP means varied by age. Younger dentists (up to 30 years) scored consistently higher than older ones (30 and more years) on items of the EE and DP factors, but not on items of the PA factor. These results corroborate findings from earlier studies [12,21,22,27] that burnout is likely to occur within the first few years of one’s career. Thus, the dentists in the older age range of our sample may be those who have survived the early stresses of their job and done well in their career. The ability to work with satisfaction over the years necessitates a knowledge and awareness of burnout, thus could underestimate burnout problems [22]. These results were in line with the variation of patterns of burnout by years of work practice experience that was presented in this study as well as was reported in other studies [22,30], although there is evidence to the contrary data [50].

Higher means of EE and DP dimensions were also significantly associated with those dentists who worked full-time. This fact deals directly with increased working hours leading to increased risk of burnout anxiety and loneliness [22,50,51]. Thus, dentists who have spent more time with patients have a higher risk to be exposed by stressors than those who work half-time. However, working time-related factors had no impact on dentist personal achievement. Many dentists in Lithuania practice in several clinics, e.g., in public clinics and the private sector, apparently due to the financial pressure, however, burnout characteristics of these dentists did not differ from those of dentists working in more than one clinic.

General dentists are the majority of the dental manpower in Lithuania, but according to the results of our study they were more likely than specialist dentists (oral surgeons, prosthodontists, orthodontists or endodontists) to report higher burnout scores in all the MBI dimensions providing a lot of significant differences in factor means. The literature review shows that examination of the burnout syndrome among dental specialists is even sparse [21,22]. Notwithstanding, it was seen that a lack of career perspectives in less qualified dentists was a major source for burnout risk [22,50,52,53]. One of the earliest studies comparing stress-induced burnout among general dentists, oral surgeons and prosthodontists was reported by Humphris et al. [54]. According to them, general dentists and oral surgeons experienced the highest levels of burnout and that prosthodontists had the lowest levels of burnout. Similar findings were reported also in recently conducted studies [55,56]. As from medical specialization, it was also learned that the risk of professional burnout is lower among specialists as compared to general practitioners [57]. Therefore, encouragement and support of dental specialty promotion programs could be recommended as a way to reduce the burnout level among dentists [22,52,58].

This study has a few limitations. First, recruiting dentists to participate in this study was conducted by in two ways, including: (i) a survey among participants of scientific conferences and (ii) an online survey. We consider that the first way ensured a randomized sampling, as only those conference participants who were selected under the precedent randomized sampling were invited to participate in the study. This subsample represented all regions of Lithuania. On the other hand, attendance of the scientific conferences is obligatory for all dentists to be able to renew their licenses, so it is unlikely that this procedure may depend on the level of professional burnout. Only one-third of the subjects in the final sample were interviewed online. This subsample cannot be considered randomized because not all dentists in the country use the Internet or Facebook account equally. There was not enough evidence to estimate a response rate. However, this proportion of subjects did not differ significantly from the former, therefore the surveyed sample could be considered as a country representative sample and to some extent this may support generalizability of the findings reported in this study.

Second, the MBI is self-reported questionnaire, so respondents’ answers may be biased. There is also an additional bias, as the survey was based on participants who voluntarily provided personal information. It is possible that those who were more prone to answer the questionnaire were also more likely to experience burnout or overestimate burnout symptoms. On the other hand, those dentists with a high level of burnout might have felt the questions too sensitive and thus been unwilling to participate in survey [48].

Third, although in our study, like in many other studies, the MBI was used to measure the burnout syndrome, caution should be employed when generalizing the results to other populations, as health care systems, cultures and populations are varied. Different health care systems may have different requirements and pressures on their dentists. Fourth, the cross-sectional design of our study limits validity of its findings, as the significant associations found in this study between burnout dimensions and other factors could not be fully explained without longitudinal studies. Finally, in the survey we collected a number of workload factors and predictors of burnout but this study was limited with analysis of several of them, so we planned to continue the analysis of the dentists’ burnout survey data in future studies.

Despite these limitations, we believe that our current findings provide further evidence regarding the MBI use in burnout syndrome research among dentists. Hence, they may have the practical benefit in planning of strategies for burnout prevention and intervention programs among dentists in Lithuania.

## 5. Conclusions

This is the first study to measure burnout syndrome among dental professionals in Lithuania. The study confirmed a three-factor model of the originally proposed Maslach burnout inventory and demonstrated its invariant structure and variance of burnout dimensions across demographic and workload groups. The presented results may contribute to lessen professional burnout among dentists in Lithuania.

## Figures and Tables

**Table 1:** Percentage distribution of respondents according to demographic and workload characteristics (*N* = 380).

Characteristics	Number (*n*)/Mean	Percentage (%)/Standard Deviation
Gender:		
Male	58	15.3
Female	322	84.7
Age (years):		
Mean and standard deviation	37.3	12.9
Up to 30	151	39.7
30 or more	229	60.3
Work practice experience (years):		
Mean and standard deviation	12.8	12.3
Up to 10 years	204	53.7
10 or more years	176	46.3
Staffing:		
Half-time	263	69.3
Full-time	117	30.7
Working in several places:		
In one clinic only	187	49.2
In two or more clinics	193	50.8
Specialty:		
Dentists in general practice	278	73.3
Oral surgeons	19	5.1
Prosthodontists	13	3.4
Orthodontists	13	3.4
Poedodontists	20	5.3
Endodontists	23	6.1
Periodontists	13	3.4

**Table 2:** Distribution of burnout levels among dentists of Lithuania under the Maslach burnout inventory (*N* = 380).

Item		Percentage Distribution On Seven-Point Likert Scale													
		Never		A Few Times a Year		Once Time a Month		A Few Times a Month		Once Time a Week		A Few Times a Week		Every Day	
		*n*	%	*n*	%	*n*	%	*n*	%	*n*	%	*n*	%	*n*	%
V1	I feel emotionally drained from my work	9	2.4	43	11.3	51	13.4	73	19.2	58	15.3	109	28.7	37	9.7
V2	I feel used up at the end of the workday	4	1.1	22	5.8	56	14.7	50	13.2	72	18.9	120	31.6	56	14.7
V3	I feel fatigued when I get up in the morning and have to face another day on the job	44	11.6	63	16.6	59	15.5	65	17.1	53	13.9	67	17.6	29	7.6
V4 ^	I can easily understand how my patients feel about things	4	1.1	4	1.1	10	2.6	19	5.0	35	9.2	87	22.9	221	58.2
V5	I feel I treat some patients as if they were impersonal objects	198	52.1	53	13.9	40	10.5	31	8.2	31	8.2	22	5.8	5	1.3
V6	Working with people all say is really a strain for me	26	6.8	53	13.9	57	15.0	60	15.8	51	13.4	71	18.7	62	16.3
V7 ^	I deal very effectively with the problems of my patients	3	0.8	4	1.1	17	4.5	34	8.9	47	12.4	130	34.2	145	38.2
V8	I feel burned out from my work	56	14.7	78	20.5	57	15.0	66	17.4	53	13.9	43	11.3	27	7.1
V9 ^	I feel I’m positively influencing other people’s lives though my work	6	1.6	6	1.6	14	3.7	19	5.0	39	10.3	101	26.6	195	51.3
V10	I’ve become more callous toward people since I took this job	165	43.4	70	18.4	37	9.7	40	10.5	31	8.2	21	5.5	16	4.2
V11	I worry that this job is hardening me emotionally	93	24.5	77	20.3	64	16.8	56	14.7	32	8.4	33	8.7	25	6.6
V12 ^	I feel very energetic	24	6.3	33	8.7	60	15.8	77	20.3	53	13.9	81	21.3	52	13.7
V13	I feel frustrated by my job	115	30.3	98	25.8	61	16.1	56	14.7	22	5.8	17	4.5	11	2.9
V14	I feel I’m working too hard on my job	118	31.1	116	30.5	57	15.0	45	11.8	19	5.0	14	3.7	11	2.9
V15	I don’t really care what happens to some patients	253	66.6	61	16.1	17	4.5	13	3.4	17	4.5	6	1.6	13	3.4
V16	Working with people directly puts too much stress on me	39	10.3	78	20.5	80	21.1	51	13.4	46	12.1	48	12.6	38	10.0
V17 ^	I can easily create a relax atmosphere with my patients	9	2.4	3	0.8	17	4.5	33	8.7	45	11.8	101	26.6	172	45.3
V18 ^	I feel exhilarated after working closely with my patients	10	2.6	15	3.9	48	12.6	67	17.6	75	19.7	106	27.9	59	15.5
V19 ^	I have accomplished many worthwhile things in this job	7	1.8	14	3.7	31	8.2	44	11.6	41	10.8	100	26.3	143	37.6
V20	I feel that this job is hardening me emotionally	85	22.4	110	28.9	49	12.9	53	13.9	44	11.6	25	6.6	14	3.7
V21 ^	In my work I deal with emotional problems very calmly	18	4.7	41	10.8	51	13.4	70	18.4	57	15.0	71	18.7	72	18.9
V22	I feel patients blame me for some of their problems	57	15.0	130	34.2	71	18.7	55	14.5	33	8.7	21	5.5	13	3.4

Note: ^ In analyses, the item was reverse-scored.

**Table 3:** Results of the exploratory factor analysis ^a^ of the Maslach burnout inventory obtained from the randomly selected subgroup 1 of the entire sample (*N* = 190).

Subscale in the Original Version [12]	Item ^b^		Four-Factor Model ^c^				Three-Factor Model ^c^		
			F1	F2	F3	F4	F1	F2	F3
	**Standardized factor loadings**								
EE	V20	I feel that this job is hardening me emotionally	**0.786**				**0.797**		
EE	V2	I feel used up at the end of the workday	**0.780**				**0.805**		
EE	V1	I feel emotionally drained from my work	**0.771**				**0.782**		
EE	V8	I feel burned out from my work	**0.759**				**0.772**		
EE	V6	Working with people all say is really a strain for me	**0.747**				**0.740**		
EE	V16	Working with people directly puts too much stress on me	**0.745**				**0.720**		
EE	V3	I feel fatigued when I get up in the morning and have to face another day on the job	**0.737**				**0.753**		
EE	V14	I feel I’m working too hard on my job	**0.663**			0.513	**0.642**		
EE	V13	I feel frustrated by my job	**0.662**			0.552	**0.654**		0.604
PA	V12	I feel very energetic	**0.608**	0.520			**0.590**	0.529	
PA	V7	I deal very effectively with the problems of my patients		**0.717**				**0.710**	
PA	V19	I have accomplished many worthwhile things in this job		**0.713**				**0.720**	
PA	V9	I feel I’m positively influencing other people’s lives though my work		**0.683**				**0.682**	
PA	V17	I can easily create a relax atmosphere with my patients		**0.672**				**0.682**	
PA	V4	I can easily understand how my patients feel about things		**0.624**				**0.626**	
PA	V18	I feel exhilarated after working closely with my patients		**0.605**				**0.602**	
PA	V21	In my work I deal with emotional problems very calmly		**0.511**				**0.504**	
DP	V10	I’ve become more callous toward people since I took this job			**0.783**		0.520		**0.684**
DP	V5	I feel I treat some patients as if they were impersonal objects			**0.696**				**0.568**
DP	V11	I worry that this job is hardening me emotionally			**0.684**		**0.535**		0.526
DP	V22	I feel patients blame me for some of their problems				**0.715**			**0.581**
DP	V15	I don’t really care what happens to some patients				**0.671**			**0.601**
	**Percentage of variances explained**		31.10	13.84	6.06	5.44	31.10	13.84	6.06
	**Cronbach’s α**		0.905	0.775	0.784	0.373	0.905	0.775	0.653

Notes: ^a^ Principal component analysis with Promax rotation (Kaiser–Meyer–Olkin measure 0.867; Bartlett’s test of sphericity *p* < 0.001). ^b^ Items are sorted by factor loadings (structure matrix) in the four-factor model. ^c^ Factor loadings greater than 0.5 are presented only; the highlighted terms indicate the main loadings for corresponding factors. EE—Emotional exhaustion; PA—Personal accomplishment; DP—Depersonalization.

**Table 4:** Estimates of three-factor model of the Maslach burnout inventory obtained from the confirmatory factor analysis of randomly selected subgroup 2 of the entire sample (*N* = 190).

	Model That Directly Replicated the EFA Results		Revised (Final) Model	
	Estimate	*p*	Estimate	*p*
Standardized Regression Weights (Factor Loadings):				
V1 ← EE	0.68	<0.001	0.66	<0.001
V2 ← EE	0.69	<0.001	0.68	<0.001
V3 ← EE	0.65	<0.001	0.66	<0.001
V6 ← EE	0.58	<0.001	0.54	<0.001
V8 ← EE	0.80	<0.001	0.82	<0.001
V14 ← EE	0.75	<0.001	0.72	<0.001
V16 ← EE	0.63	<0.001	0.57	<0.001
V20 ← EE	0.77	<0.001	0.80	<0.001
V4 ← PA	0.47	<0.001	0.45	<0.001
V7 ← PA	0.64	<0.001	0.56	<0.001
V9 ← PA	0.60	<0.001	0.52	<0.001
V17 ← PA	0.65	<0.001	0.63	<0.001
V18 ← PA	0.62	<0.001	0.66	<0.001
V21 ← PA	0.50	<0.001	0.53	<0.001
V19 ← PA	0.71	<0.001	0.75	<0.001
V5 ← DP	0.59	<0.001	0.61	<0.001
V15 ← DP	0.46	<0.001	0.46	<0.001
V22 ← DP	0.47	<0.001	0.45	<0.001
V12 ← EE	0.45	<0.001	0.44	<0.001
V12 ← PA	0.25	0.001	0.28	<0.001
V13 ← EE	0.57	<0.001	0.58	<0.001
V13 ← DP	0.27	0.002	0.22	0.001
V10 ← EE	0.21	0.161	-	
V10 ← DP	0.98	<0.001	0.90	<0.001
V11 ← EE	−0.05	0.679	-	
V11 ← DP	0.82	<0.001	0.80	<0.001
**Model fit estimates:**				
Chi-squared/df	2.36	<0.001	1.67	<0.001
CFI	0.85		0.93	
TLI	0.82		0.92	
IFI	0.85		0.93	
RMSEA (90% CI)	0.085 (0.075–0.095)		0.059 (0.048–0.070)	
**Correlations:**				
DP ←→ PA	0.41	<0.001	0.43	<0.001
PA ←→ EE	0.38	<0.001	0.39	<0.001
DP ←→ EE	0.72	<0.001	0.65	<0.001

Notes: EE—emotional exhaustion; PA—personal accomplishment; DP—depersonalization; EFA—exploratory factor analysis; df—degree of freedom; CFI—comparative fit index; TLI—Tucker–Lewis index; IFI—Bollen’s incremental fit index; RMSEA—root mean square error of approximation; CI—confidence interval; V1, ..., V22 are the MBI items.

**Table 5:** Testing the final factor model’s invariance across subsamples, gender, age and staffing groups (*N* = 380).

Compared Study Groups	Constraints	χ^2^	df	χ^2^/df	CFI	TLI	IFI	RMSEA (90% CI)
Subsamples: 1 vs. 2	Identical path diagrams	659.3	398	1.66	0.92	0.91	0.92	0.042 (0.036–0.047)
	Constant factor loadings	682.5	419	1.63	0.92	0.91	0.92	0.041 (0.035–0.046)
	Constant factor variances and covariances	695.8	425	1.64	0.92	0.91	0.92	0.041 (0.035–0.046)
Gender: males vs. females	Identical path diagrams	693.4	398	1.74	0.92	0.90	0.92	0.044 (0.039–0.050)
	Constant factor loadings	720.9	419	1.72	0.91	0.90	0.92	0.044 (0.038–0.049)
	Constant factor variances and covariances	730.6	425	1.72	0.91	0.91	0.91	0.044 (0.038–0.048)
Age (years): Up to 30 vs. 30 or more	Identical path diagrams	743.6	398	1.87	0.90	0.89	0.91	0.048 (0.043–0.054)
	Constant factor loadings	794.0	419	1.90	0.90	0.89	0.90	0.049 (0.044–0.054)
	Constant factor variances and covariances	803.3	425	1.89	0.90	0.89	0.90	0.049 (0.044–0.054)
Staffing: half-time vs. full-time	Identical path diagrams	712.9	398	1.79	0.91	0.89	0.91	0.046 (0.041–0.052)
	Constant factor loadings	738.5	419	1.76	0.91	0.90	0.91	0.045 (0.040–0.051)
	Constant factor variances and covariances	745.8	425	1.76	0.91	0.90	0.91	0.045 (0.040–0.051)

Notes: df—degree of freedom; CFI—comparative fit index; TLI—Tucker–Lewis index; IFI—Bollen’s incremental fit index; RMSEA—root mean square error of approximation; CI—confidence interval; V1, ..., V22 are the MBI items.

**Table 6:** Comparison of mean values of the Maslach burnout inventory common factors between demographic and workload groups (*N* = 380).

Compared Study Groups			Difference in Means Between Group 2 And Group 1								
Variable	Group 1	Group 2	EE			DP			PA		
			Estimate	SE	*p*	Estimate	SE	*p*	Estimate	SE	*p*
Gender	Male	Female	0.386	0.159	**0.015**	0.157	0.141	0.265	0.003	0.145	0.983
Age	Up to 30 years	30 or more years	−0.381	0.127	**0.003**	−0.422	0.124	**<0.001**	−0.073	0.027	0.796
Work practice experience	Up to 10 years	10 or more years	−0.395	0.130	**0.002**	−0.463	0.118	**<0.001**	−0.140	0.106	0.186
Staffing	Half-time	Full-time	0.515	0.134	**<0.001**	0.420	0.131	**0.001**	0.026	0.112	0.819
Working in several places	In one clinic only	In two or more clinics	0.166	0.124	0.182	0.082	0.119	0.490	−0.067	0.106	0.528
Specialization of dentists	Dentists in general practice	Oral surgeons	−0.776	0.254	**0.002**	−0.445	0.219	**0.002**	−0.226	0.235	0.336
		Prosthodontists	−0.565	0.328	0.085	−0.693	0.183	**<0.001**	−0.491	0.246	**0.046**
		Orthodontists	−0.583	0.204	**0.004**	−0.804	0.145	**<0.001**	−0.012	0.307	0.968
		Poedodontists	−0.319	0.284	0.262	−0.440	0.227	0.053	−0.429	0.227	0.059
		Endodontists	−0.864	0.216	**<0.001**	−0.635	0.188	**<0.001**	−0.240	0.219	0.273
		Periodontists	0.152	0.298	0.610	0.216	0.433	0.617	0.060	0.288	0.834

Notes: EE—emotional exhaustion, DP—depersonalization, PA—personal accomplishment. SE—standard error. *p*-values < 0.05 are highlighted.

## Abbreviations

The following abbreviations were used in this manuscript:

CFA	Confirmatory factor analysis
CFI	Comparative fit index
CI	Confidence interval
DP	Depersonalization
EE	Emotional exhaustion
EFA	Exploratory factor analysis
IFI	Bollen’s incremental fit index
KMO	Kaiser-Meyer-Olkin measure
MBI	Maslach Burnout Inventory
PA	Personal accomplishment
RMSEA	Root mean square error of approximation
SD	Standard deviation
TLI	Tucker–Lewis index
